# n-3 PUFA Enriched Eggs as a Source of Valuable Bioactive Substances

**DOI:** 10.3390/foods12234202

**Published:** 2023-11-22

**Authors:** Ana Radanović, Gordana Kralik, Ines Drenjančević, Olivera Galović, Manuela Košević, Zlata Kralik

**Affiliations:** 1 Agro-Kovačević, Vijenac Dinare 2, 31000 Osijek, Croatia; azelicos@gmail.com; 2 Nutricin j.d.o.o. Darda, Braće Radića 6, 31326 Darda, Croatia; gkralik@fazos.hr; 3 Scientific Center of Excellence for Personalized Health Care, Josip Juraj Strossmayer University of Osijek, Trg sv. Trojstva 3, 31000 Osijek, Croatia; idrenjancevic@mefos.hr (I.D.); ogalovic@kemija.unios.hr (O.G.); manuela.kosevic@fazos.hr (M.K.); 4 Department of Physiology and Immunology, Faculty of Medicine, Josip Juraj Strossmayer University of Osijek, Josipa Huttlera 4, 31000 Osijek, Croatia; 5 Department of Chemistry, Josip Juraj Strossmayer University of Osijek, Cara Hadrijana 8a, 31000 Osijek, Croatia; 6 Faculty of Agrobiotechnical Sciences Osijek, Josip Juraj Strossmayer University of Osijek, V. Preloga 1, 31000 Osijek, Croatia

**Keywords:** eggs, n-3 PUFA, consumption, blood biochemical indicators

## Abstract

This research elaborates the process of enriching table eggs with n-3 polyunsaturated fatty acids (n-3 PUFA) and presents the effect of such enriched eggs on human health. The experiment was performed on 480 TETRA SL laying hens divided into three groups. Feeding mixtures contained 5% of oils (K = soybean oil, P1 = 3.5% linseed oil + 1.5% fish oil, P2 = 3% linseed oil + 2% fish oil). Referring to the content of α-linolenic acid (ALA), eicosapentaenoic acid (EPA), and docosahexaenoic acid (DHA), eggs of P1 and P2 groups were significantly richer in n-3 PUFA than eggs of the control group (*p* = 0.001). Atherogenic (AI), thrombogenic (TI), and hypo/hypercholesterolemic (HHI) indexes of egg yolks were more favourable in enriched eggs than in conventional eggs. Fatty acid profiles in the blood of examinees that consumed conventional and enriched eggs (treatments K and P1, respectively) differed significantly in total saturated fatty acids (ΣSFA) (*p* = 0.041) and in the content of ALA (*p* = 0.010). The consumption of n-3 PUFA-enriched eggs lowered the Σn-6 PUFA/Σn-3 PUFA ratio in the examinees’ blood serum (27%) and had a favourable effect on some blood biochemical indicators. This research confirmed the assumption that the use of a combination of fish and linseed oil in mixtures for laying hens in an amount of up to 5% will increase the content of omega-3 in table eggs, but it was not confirmed that the consumption of these eggs in a short period of time (21 days) has a positive effect on human health.

## 1. Introduction

Eggs are an excellent source of nutrients for humans and are considered a readily available source of protein around the world. In addition to proteins, they also contain many substances with an important biological function. The composition of fatty acids in conventional eggs is relatively unfavourable. Of the total fatty acids in yolk lipids, saturated fatty acids (SFA) make up 30–35%, monounsaturated fatty acids (MUFA) make up 40–45%, whereas the least represented (20–25%) are polyunsaturated fatty acids (PUFA) [1]. Eggs of laying hens fed standard feed mixtures contain a higher proportion of n-6 PUFA and a lower proportion of n-3 PUFA [2]. Precisely because of the unfavourable composition of fatty acids in conventional eggs, recent studies are focused on investigating the possibilities of enriching table eggs with n-3 polyunsaturated fatty acids (n-3 PUFA) to make them a natural source of that nutricine. It is possible to increase the levels of n-3 PUFA in eggs by feeding laying hens with feed mixtures with the addition of rapeseed, linseed, and fish oil. Linseed oil in its profile contain a higher proportion of α-linolenic fatty acid (ALA) content, which is essential for humans, whereas fish oil is rich in eicosapentaenoic (EPA) and docosahexaenoic (DHA) fatty acids [3]. As a human organism cannot synthesise essential fatty acids, they have to be taken in through food [4]. In their research, many authors designed feed mixtures for laying hens in order to increase the content of ALA, EPA, and DHA in eggs and reduce the n-6 PUFA/n-3 PUFA ratio [1,5,6]. Promila et al. [7] used mixtures containing 1–4% linseed oil in the feeding of laying hens. The content of ALA increased from 1.52% to 9.79% in total fatty acids. Yalcin and Unal [5] used 4.5% fish oil as well as a combination of linseeds and fish oil (10% + 1.5%) in the feeding of laying hens. After 30 days of feeding with the mentioned treatments, they found in yolk lipids 4.10% ALA, 0.55% EPA, and 3.91% DHA, i.e., a total of 9.72% ∑n-3 PUFA, and the ratio n-6/n-3 PUFA was 2.28. The use of fish oil in mixtures for laying hens in concentrations of 0.3–1.5%, in combination with soybean oil (up to 5% in total), resulted in an increase in n-3 PUFA from 258.44 to 327.35 mg/100 g of eggs [8]. When using fish oil in the feeding of laying hens, ALA can increase in eggs 4 to 8 times and ∑n-3 PUFA 2 to 6 times [6,9]. However, when a higher percentage of fish oil, but also linseed oil, is added to chicken mixtures, the eggs have a fishy smell that is difficult for consumers to accept [3]. Except for the sensory properties, the addition of the mentioned oils in mixtures for laying hens can affect the number but also the quality of n-3 PUFA eggs [3,10]. Lawlor et al. [10] in their research used mixtures with 0%, 2%, 4%, and 6% fish oil and in conclusion pointed out that hard-boiled eggs from the treatment with the addition of 6% fish oil had a significant (*p* < 0.001) more intense sulphur flavour compared to the eggs of the other tested treatments. The addition of 2 to 3% of linseed oil to the diet of laying hens increases the omega-3 PUFA content and does not cause significant changes in production performance and egg quality [6]; however, feed containing 5% or more linseed oil can affect the quality of the eggshell [11]. The modern human diet does not meet the daily requirements for n-3 polyunsaturated fatty acids [12].

The interest in the effects of n-3 PUFA on human health emerged during the 1970s, after Danish researchers discovered that Eskimos in Greenland had a low incidence of cardiovascular diseases despite eating food rich in saturated fatty acids. A research study managed to prove that such positive health status was affected by the dietary intake of long-chain n-3 PUFA, i.e., by consumption of significant amount of fish (oil) and seafood [13]. Berlin et al. [14] stated that the ideal ratio of n-6 PUFA to n-3 PUFA intake for humans would be between 4:1 and 10:1. It has been proven that n-3 PUFA affect the reduction in blood triglyceride levels, blood pressure, prevent type 2 diabetes, cardiovascular system diseases, and some forms of cancer [15,16]. Human organisms absorb two fatty acids, linoleic and α-linolenic, from food to form initial metabolites needed for the synthesis of other important fatty acids. Linoleic fatty acid is metabolised into arachidonic fatty acid, and α-linolenic acid is metabolised into eicosapentaenoic and docosahexaenoic fatty acids. The metabolic pathways of n-6 PUFA and n-3 PUFA consist of alternating desaturation and elongation reactions, by which desaturation enzymes introduce a new double bond into the carbon chain, whereas elongation enzymes add two new C atoms. After the synthesis, PUFA are stored in an esterified form in phospholipids or as neutral glycerides. If necessary, through action of cyclooxygenase and lipoxygenase enzymes, PUFA serve to create bioactive signalling molecules of eicosanoids [15,17]. As there are very few studies covering the process of omega-3 egg production, consumption by consumers, and their impacts on human health, the objective of this research was to produce eggs enriched with n-3 PUFA, with the hypothesis that consumption of those eggs will contribute to a significant intake of n-3 PUFA and induce positive effects on human health status. 

## 2. Materials and Methods

### 2.1. Housing and Feeding of Laying Hens

Experimental treatments of laying hens and analysis of produced eggs used in this research were aligned with the protocols approved by the Animal Research Ethics Committee of the Faculty of Agrobiotechnical Sciences Osijek. The experiment was performed on 480 laying hens of the TETRA SL hybrid, which were divided into three groups. Each group consisted of 160 laying hens further divided into 16 repetitions. Laying hens of the control group K consumed diet with 5% soybean oil, the experimental group P1 was given diet supplemented with a combination of 1.5% fish oil and 3.5% linseed oil, and the experimental group P2 had diet supplemented with 2% fish oil and 3% linseed oil. Soybean oil was purchased from Zvijezda plus d.o.o. (Zagreb, Croatia), linseed oil from Nutrifit d.o.o. (Zagreb, Croatia), and unrefined fish oil was obtained from factory Sadrina d.o.o. (Postira, Croatia). Feeding mixtures for laying hens were balanced at 17.00% crude protein and 12.10 MJ/kg ME. Experimental period lasted for 30 days. After 21st day, eggs were sampled for the analysis of the fatty acid profile. During the remaining 7 days, eggs were collected to be used in experiment performed on examinees. Laying hens were offered feed and water ad libitum. Composition of feeding mixtures, i.e., diets fed to laying hens used in the experiment is overviewed in Table 1.

Laying hens were kept in cages, with a disposable area of 750 cm^2^ per hen, of which 600 cm^2^ was usable area. Cages with hens were placed in one poultry house to assure equal microclimatic conditions. Applied lighting regime was 16 h of light and 8 h of darkness. 

### 2.2. Analysis of the Fatty Acid Profile in Feeding Mixtures and in Egg Yolks

The analysis was performed on a sample of 30 eggs and on samples of 3 laying hens’ feeding mixtures. The fatty acid profile was determined on 30 yolks, i.e., on 10 yolks sampled from each group. Samples of feeding mixtures were analysed in three parallel runs. The sample for determination of the fatty acid profile was prepared according to the standard method of fat extraction and the fatty acid hydrolysis and esterification. Content of fatty acids was determined according to the method of Csapó et al. [18]. The fatty acid profile was determined in a SCION 436-GC gas chromatograph (SCION Instruments), equipped with FAMEWAX capillary column (RESTEK, Bellefonte, PA, USA), with inner diameter of 30 × 0.32 mm, film of 0.25 μm, and a flame ionization detector. The sample volume for injection was 1 μL, and the operating conditions were as follows: injector temperature 230 °C, detector temperature 230 °C, and carrier gas flow (hydrogen) 2.5 mL/min. The oven heating was set as follows: from 50 to 160 °C: 20 °C/min; from 160 to 225 °C: 10 °C/min, with nine minute-hold at 225 °C. The total duration of the analysis was 21 min. A standard mixture of 37 fatty acids (Food Industry FAME Mix, Restek Corporation, Bellefonte, PA, USA) was used to determine the individual fatty acids. Contents of individual fatty acids in the feeding mixture samples are presented as a percentage of total fatty acids in the lipids, whereas the content of individual fatty acids in egg yolks is expressed in mg/100 g of yolk.

The fatty acid profile was determined on fresh eggs and not on boiled eggs because eggs for volunteers were prepared according to the protocol from the paper Kralik et al. [19], where it was determined that boiling did not significantly reduce the content of fatty acids in eggs.

### 2.3. Calculation of Atherogenic, Thrombogenic and Hypo/Hypercholesterolemic Indexes

Based on the analysis of fatty acids in yolk lipids, the following egg health indexes were calculated:

- atherogenic index (AI);
- thrombogenic index (TI);
- hypo/hypercholesterolemic index (HHI).

AI and TI are calculated according to Omri et al. [20], whereas HHI is calculated according to Fernandez et al. [21] by applying the following formulas:

AI = (4 × C14:0 + C16:0 + C18:0)/(∑ MUFA + ∑ n-6 PUFA + ∑ n-3 PUFA)(1)

TI = (C14:0 + C16:0 + C18:0)/0. ×∑ MUFA+ 0.5×∑ n-6 PUFA + 3×∑ n-3 PUFA + (∑ n-3 PUFA/∑ n-6 PUFA)(2)

HHI = (C18:1 n-9 + C18:2 n-6 + C20:4 n-6 + C18:3 n-3 + C20:5 n-3 + C22:5 n-3 + C22:6 n-3)/(C14:0 + C16:0)(3)

where MUFA = monounsaturated fatty acids, and PUFA = polyunsaturated fatty acids. Fatty acid C22:5 n-3 was not detected in groups K, P1, and P2, and C20:5 n-3 was not detected in eggs of the K group.

### 2.4. Selection of Examinees, Analysis of Blood Indicators

After gaining insight into the efficiency of egg enrichment with n-3 PUFA and calculation of egg health indexes, there were two groups of laying hens (K and P1) selected for further laying of eggs to be consumed by examinees. Although no significant differences in the fatty acid profile were found between the P1 and P2 groups, more favourable n-3 PUFA values as well as a more favourable n-6/n-3 PUFA ratio were observed in the P1 group. For this reason, it was not justified to test both experimental groups of enriched eggs on examinees. Therefore, the P1 group was selected for further research. Examinees were involved in a randomized, double-blind study (registration at ClinicalTrials, https://clinicaltrials.gov/ (accessed on 17 January 2022), identification: NCT02720250). There were 20 young healthy examinees aged 18–30 with normal body mass index (BMI) selected for the experiment. Before selecting the examinees, the criterion of exclusion was employed. Exclusion criteria were smoking, hypertension, coronary artery disease, diabetes, hyperlipidemia, renal impairment, cerebrovascular and peripheral arterial disease, and intake of any medication or substance that could affect the endothelium. Furthermore, none of the examinees consumed n-3 PUFA-enriched functional food or n-3 PUFA dietary supplements before joining this experiment. Once the selected examinees met all criteria, they were familiarised with the research protocol and procedures that followed the standards set by the last revision of the Declaration of Helsinki. Research procedures were approved by the Ethics Committee of the Faculty of Medicine of the University of Osijek (Class: 602-04/14-08/06; Ref. No.: 2158-610714-114). Each examinee signed a consent to the participation in this research. Over a period of 21 days, each examinee consumed 63 boiled eggs in total, i.e., three boiled eggs daily. In the Republic of Croatia, the Rulebook on market standards for eggs (Official Gazette 90/2021) [22] is in force. According to this Rulebook, eggs intended for sale and consumption are classified into 4 grades according to weight (S < 53 g; M from 53 g to 63 g; L from 63 g to 73 g; and XL > 73 g). Given that during the production cycle, hens lay the most M and L grade eggs, which are constantly available to our consumers on the market, in this research, we chose the L grade eggs for testing on examinees. The control group of examinees (K) consumed eggs laid by the control group of laying hens. Experimental group of examinees (P1) consumed eggs laid by the P1 group of laying hens that were fed diet with an increased content of n-3 PUFA. Control group of examinees consumed 264 mg of n-3 PUFA per day, whereas experimental group of examinees ate eggs that contained 1051.60 mg of n-3 PUFA per day. Neither scientists nor examinees had information to which group the examinees were assigned until the end of the three-week dietary protocol (eggs were labelled #1 or #2 before being distributed to the Laboratory). The research involving examinees was conducted by the Laboratory for Clinical and Sports Physiology of the Department of Physiology and Immunology at the Faculty of Medicine in Osijek. Before and after the implemented dietary protocol, examinees were subjected to tests in the morning after overnight fasting. Prior to blood testing, examinees were instructed not to undertake any strenuous activity for 24 h before and to avoid caffeine intake the morning before the blood sampling. A blood sample was taken from a vein of each examinee 15 min after they rested in a sitting position. The following indicators were analysed from the blood: leukocytes, erythrocytes, haemoglobin, haematocrit, MCV (Mean Corpuscular Volume), MCH (Mean Corpuscular Haemoglobin), MCHC (Mean Corpuscular Haemoglobin Concentration), RDW-CV (Red blood cell Distribution Width), platelets, MPV (Mean Platelet Volume), erythrocyte sedimentation rate, fibrinogen, glucose, urea, creatinine, urate, hsCRP (High Sensitivity C-Reactive Protein), iron, transferrin, cholesterol, triglycerides, HDL and LDL cholesterol, and HDL-C/cholesterol.

### 2.5. Statistical Data Analysis

The TIBCO Statistica Software version 13.5.0. [23] was used to process data on the content of fatty acids in egg yolks. The data were used to determine the GLM procedure, analysis of variance, and to run the Fisher’s LSD test to determine significant differences at levels *p* ˂ 0.05; *p* ˂ 0.01 and *p* ˂ 0.001. Results of blood sample tests were analysed in Excel, and differences between groups were tested with the student’s *t*-test. Results are presented in tables below as arithmetic mean ($\bar{x}$) and standard deviation (sd). 

## 3. Results and Discussion

### 3.1. Content of Fatty Acids in Laying Hens’ Feeding Mixtures

Table 2 presents results of the fatty acid content in feeding mixtures fed to laying hens of the control (K) and experimental groups (P1 and P2). Feeding mixture fed to the control group (K) contained 16.72% saturated fatty acids (SFA), 26.47% MUFA, 51.84% n-6 PUFA, and 5.04% n-3 PUFA. The ratio n-6/n-3 PUFA of the K feeding mixture was 10.28:1. The content of EPA and DHA was not detected in the K feeding mixture. Experimental feeding mixtures fed to the P1 and P2 group contained 16.84% and 17.33% SFA, 25.89% and 26.28% MUFA, and 23.40% and 22.79% n-6 PUFA, respectively. The content of n-3 PUFA in experimental feeding mixtures was similar, being 33.89% in P1 and 33.59% in P2. The ratio of n-6/n-3 PUFA in the P1 feeding mixture was 0.69:1, and it was 0.68:1 in P2. The content of EPA and DHA in P2 feeding mixture increased from 1.86% and 2.42% to 3.53% and 4.84%, respectively. There were differences (*p* = 0.001) determined with respect to n-3 PUFA and the ratio of n-6/n-3 PUFA between the control and experimental groups (*p* = 0.001). Results of feed analyses are in accordance with results published by other authors [5,24]. 

### 3.2. Content of Fatty Acids in Egg Yolk Lipids

Table 3 overviews the profile of fatty acids (mg/100 g) in egg yolks of the groups K, P1, and P2. Chemical analysis of the ∑SFA confirmed significant differences between groups (*p* = 0.036). There were also significant differences determined between groups with respect to the contents of the following fatty acids: myristic (C14:0; *p* = 0.001), pentadecanoic (C15:0; *p* = 0.002), palmitic (C16:0; *p* = 0.005), and heneicosanoic (C21:0; *p* = 0.001). The content of ∑MUFA differed significantly between eggs of the groups K, P1, and P2 (*p* = 0.001). The P1 and P2 eggs contained significantly more myristoleic (C14:1; *p* = 0.001), palmitoleic (C16:1; *p* = 0.001), and cis-10-heptadecenoic (C17:1; *p* = 0.005) acids. When compared to the control, the contents of ∑n-6 PUFA in egg yolk lipids of the experimental P1 and P2 groups were significantly lower (*p* = 0.002). The contents of linoleic (C18:2, n-6; *p* = 0.002) and arachidonic acids (C20:4, n-6; *p* = 0001) were also reduced. Analysis of the contents of ALA, EPA, and DHA confirmed that eggs of the P1 and P2 groups were significantly richer in n-3 PUFA than eggs of the control (*p* = 0.001). The contents of alpha-linolenic acid (C18:3, n-3) in the edible parts of the eggs of the control were 65.48 mg/100 g, whereas the same contents in edible part of eggs of P1 and P2 group were 370.27 and 358.21 mg/100 g, respectively, (*p* = 0.001). Eggs of the control did not contain EPA (C20:5, n-3), whereas EPA in edible part of eggs of P1 and P2 group amounted to 29.41 and 31.06 mg/100 g, respectively (*p* = 0.001). There was significantly more DHA (C22:6, n-3) contained in the eggs of the P1 and P2 groups (199.18 and 191.44 mg/100 g, respectively) than in the control (84.40 mg/100 g of egg; *p* = 0.001). Total content of ∑n-3 PUFA in the control eggs was 149.88, in P2 eggs it was 580.71 mg/100 g of egg, whereas in P1 eggs it amounted to 598.59 mg/100 g of egg. Results of this research prove that egg yolk lipids of the control (conventional) eggs contained significantly less ∑n-3 PUFA than eggs of experimental P1 and P2 groups, which was in accordance with results published by Nimalaratne and Wu [1], who also investigated enrichment of eggs with n-3 PUFA.

When compared to the experimental groups, eggs laid by hens in the control being fed the standard diet contained more ∑n-6 PUFA and less ∑n-3 PUFA. The same finding was also reported by Khan et al. [25]. This research result is aligned with the results published by Kralik et al. [8], who argued that the content of fatty acids in egg yolks could be modified through supplementation of n-3 PUFA-rich ingredients to laying hens’ feed. Consumption of linseed oil and fish oil supplemented to the diet of the P1 group (3.5% linseed oil + 1.5% fish oil) and of the P2 group (3% linseed oil + 2% fish oil) affected the metabolic process in hens, by which n-3 PUFA was deposited in yolk lipids, thus enriching eggs with n-3 PUFA. Consequently, the ratio of ∑n-6/n-3 PUFA in P1 eggs was 1.88:1, and in P2 eggs it was 1.98:1, whereas in eggs of the control group that ratio was 10.36:1. Such research results correspond with those published by Alagawany et al. [24] and Kralik et al. [26]. Yalcin and Unal [5] supplemented laying hens’ feed with 4.5% fish oil and with a combination of fish oil and linseeds (1.5% + 10%) to accomplish 9.72% of ∑n-3 PUFA and the ratio ∑n-6/n-3 PUFA of 2.28:1. 

### 3.3. Egg Lipid Indexes

When defining indicators of lipid quality for human health, fatty acids are classified according to their structural formulas (SFA, MUFA, PUFA). There are also atherogenic (AI) and thrombogenic (TI) indexes used, as well as the index referring to the ratio of hypocholesterolemic and hypercholesterolemic fatty acids (HHI) [20,27]. In Table 4, there is an overview of quality indicators in egg yolk lipids as measured in this research. Having in mind their benefits on human health, the qualities of lipids contained in egg yolks in this research are presented by AI, TI, and HHI. Values of AI and TI were the greatest in eggs of the control (0.498 and 0.833, respectively), whereas the same values were lower in eggs of P1 and P2 groups (0.471 and 0.470, and 0.555 and 0.556, respectively). AI shows the ratio of the main saturated and unsaturated fatty acids. It is considered that unsaturated fatty acids have anti-atherogenic effects, as they inhibit the aggregation of plaques, and they contain less esterified fatty acids, cholesterol, and phospholipids, thereby preventing the occurrence of coronary diseases.

Thrombogenic index shows the ratio of pro-thrombogenic (saturated) and anti-thrombogenic (unsaturated) fatty acids [20]. Atherogenic index is significant because it includes, along with MUFA, myristic fatty acid (C14:0), which is considered to have the most harmful cardiovascular effects [28]. TI indicates a risk of blood clotting. HHI show effects of certain fatty acids involved in cholesterol metabolism, and higher values are preferred. Consumption of certain fatty acids can either stimulate or prevent atherosclerosis and thrombosis, which are influenced by the content of total cholesterol and LDL cholesterol [29]. It is considered that AI and TI can show the atherogenic and thrombogenic potential of egg yolk lipids better than the PUFA/SFA ratio. Furthermore, AI and TI show to what extent individual fatty acids affect the increase in pathogenic risk of atheroma and/or thrombi that directly affect human health. Eggs with lower SFA/PUFA ratios exhibit lower AI, TI, and HHI [20,27,30]. El-Wakf et al. [31] reported that consumption of eggs with lower AI and TI reduced the risk of atherosclerosis, and Hosseini-Vashan et al. [32] and Watson et al. [33] stated that low TI reduced atrial fibrillation. Myristic and palmitic fatty acids are considered as atherogenic, yet some authors argue that palmitic fatty acid was neutral in terms of atherogenicity, and they attribute thrombogenic properties to it [32,34,35].

### 3.4. Fatty Acid Profile in Examinees’ Blood Serum 

Table 5 overviews the fatty acid profiles obtained from blood serum of examinees that consumed either conventional eggs (K group) or eggs enriched with n-3 PUFA (P1 group). 

A significant difference was determined in the content of palmitic fatty acid (C16:0; *p* = 0.031), as well as in the total saturated fatty acids (∑SFA, *p* = 0.041). Difference in ∑MUFA in blood serum between K and P1 group of examinees was not significant (*p* = 0.130). However, significant difference was confirmed for the content of dichomo-gamma-linolenic acid (C20:3, n-6; *p* = 0.012) and arachidonic acid (C20:4, n-6; *p* = 0.031). Serum of the K group contained more ∑n-6 PUFA (*p* > 0.142) than the serum of the P1 group. While comparing the contents of specific n-3 PUFA, significant difference between the examinees’ groups was confirmed only in the content of ALA (*p* = 0.010). Although the serum of P1 group exhibited higher content of ∑n-3 PUFA than the serum of the K group (71.95:65.44), this difference was not relevant (*p* = 0.861). We assume that this difference is not significant due to the short period of consumption of enriched eggs as well as the small number of examinees, although the eggs of the P1 group that were used in the study had a very high content of n-3 PUFA compared to the eggs of the control group. From the nutritional aspect, in addition to the ALA, EPA, and DHA concentrations, as well as ∑n-3 PUFA, the ratio of ∑n-6 PUFA/∑n-3 PUFA is also important. In this research, such a ratio was 22.44:1 in the K group, which became reduced to 16.11:1 in the P1 group of examinees. Lowering of the mentioned ratio by 27% occurred because of the beneficial effect that consumption of n-3 PUFA-enriched eggs had on human organisms. Such research results agree with the results published by Stupin et al. [36]. Some experiments established a connection between the frequency of cardiovascular diseases and a reduced portion of n-3 PUFA in the blood serum, by which a strong relation was established between the reduced portion of EPA and AA (EPA/AA) in the serum and the appearance of acute coronary symptoms, as well as high prevalence of complex coronary lesions [37,38,39]. However, a modern Western diet is rich in n-6 PUFA and deficient in n-3 PUFA [40].

### 3.5. Arterial Pressure and Biochemical Indicators in Examinees’ Blood

In Table 6, there are data on measured arterial blood pressure of examinees. As presented, the blood pressure levels of examinees in both groups (K and P1) were within the optimal limits (<120/80 mm Hg), which corresponded to the examinees’ profile since the experiment was conducted on young healthy individuals. The obtained research results did not establish significant differences in blood pressure between examinees who consumed either conventional eggs or n-3 PUFA-enriched eggs. Furthermore, there were no significant differences in the blood pressure values measured at the experiment’s start and at the end of 21-day long experimental period, during which examinees consumed 3 boiled eggs per day. Our results are in accordance with the results reported by Stupin et al. [36], who performed research on young and healthy individuals and found no significant differences in the measured values of SBP (Systolic Blood Pressure; mm Hg), DBP (Diastolic Blood Pressure; mm Hg), and MAP (Mean Arterial Pressure; mm Hg) between the control group that had been given conventional eggs at the daily intake of 249 mg n-3 PUFA and the experimental group that consumed eggs enriched with n-3 PUFA (1053 mg n-3 PUFA/day), except for the values of systolic blood pressure of the control, as their SBP was significantly lower at the end of the experimental period than at the start of the experiment (*p* < 0.05).

The same results were reported by Mihalj et al. [41] in their experiment on young, healthy persons. They obtained similar values of arterial blood pressure at the beginning and at the end of experiment in both groups that consumed two boiled eggs per day over three weeks (control group—75 mg n-3 PUFA/day; n-3 PUFA group—470 mg n-3 PUFA/day). 

Table 7 presents blood biochemical indicators for the control group (K, n = 10) that consumed conventional eggs and for the experimental group (P1, n = 10) that consumed eggs enriched with n-3 PUFA. Those results refer to blood biochemical indicators measured before and after the egg consumption period. The leukocytes, erythrocytes, haemoglobin, haematocrit, MCV (Mean Corpuscular Volume), MCH (Mean Corpuscular Haemoglobin), MCHC (Mean Corpuscular Haemoglobin Concentration), RDW-CV (Red blood cell Distribution Width), and MPV (Mean Platelet Volume) of both groups were within normal values. The level of hsCRP (High Sensitivity C-Reactive Protein) was lower in examinees that consumed n-3 PUFA eggs (1.34 mg/L) than in examinees that consumed conventional eggs (3.4 mg/L).

It was also observed that iron was higher in the P1 group than in the K group at the end of the experiment (20.55:13.00, respectively), although both values were in the reference range. Levels of cholesterol, triglycerides, HDL cholesterol, and LDL cholesterol in both groups were also normal. These results were consistent with results published by other authors [36,42]. Dietary cholesterol takes up, on average, one third of total body cholesterol, whereas the rest is synthesised in the organism [43]. High blood cholesterol levels point out the risk of cardiovascular diseases in humans. Cholesterol is involved in creation of cell membranes, in synthesis of sex hormones, as well as adrenal gland hormones and vitamin D. It is also a precursor of bile acids. LDL cholesterol (low-density lipoproteins) transports cholesterol from the liver to the cells, and the surplus is deposited on the walls of the arteries, thus forming plaque that can cause clogging of blood vessels. HDL cholesterol (high-density lipoproteins) collects the surplus of cholesterol from the blood and tissues and transports it to the liver. This reduces the risk of cardiovascular diseases [44].

The presented research did not confirm any significant differences in the blood serum lipid profile in examinees who consumed n-3 PUFA-enriched eggs. Such a finding is consistent with previous studies, which argued that EPA and DHA affected the reduction in triglycerides (9–26%) if they were consumed in large amounts (>4 g/day) contained in functional products [45]. Bovet et al. [46] reported that the consumption of five n-3 PUFA-enriched eggs (241 mg of n-3 PUFA/egg) during 3 weeks significantly reduced triglycerides level in blood serum (16–18%) in healthy individuals. Other studies [47,48] also confirmed that consumption of n-3 PUFA-enriched eggs could potentially reduce triglycerides in blood serum of healthy individuals. Jacobson et al. [49] and Kris-Etherton et al. [50] stated that food containing increased amount of EPA and DHA could reduce the concentration of triglycerides, which was also confirmed by our research (*p* > 0.05%). Clinical studies and meta-analyses performed on hyperlipidemic individuals report that dietary supplementation with EPA and DHA could reduce lipid concentrations, mainly triglycerides in human blood serum [51].

## 4. Conclusions

Based on the results obtained within the study into enrichment of table eggs with n-3 PUFA and performed bioclinical tests on examinees, the following conclusions are reached:

Fatty acid profiles in egg lipids depended on the feeding treatment of laying hens. The concentrations of ALA, EPA, and DHA as well as the n-6/n3 PUFA ratio are more favourable in the eggs of groups P1 and P2 compared to group K (*p* < 0.05). The quality of yolk lipids was presented by atherogenic, thrombogenic, and hypo/hypercholesterolemic indexes, which were in favour of the groups P1 and P2, when compared to the K group. After having consumed conventional eggs (group K) and n-3 PUFA-enriched eggs (group P1), the fatty acid profile in examinees’ blood differed significantly between K and P1 group with respect to ∑SFA (*p* = 0.041) and ALA content (*p* = 0.010). The ratio of ∑n-6/n-3 PUFA was lowered by 27.23%, which is considered favourable from the aspect of nutritional benefit. The analysis of examinees’ blood biochemical indicators showed that the P1 group had lower values of MCV (fl), MVP (f), and urates (*p* < 0.05) and higher values of MCHC (g/L) after having consumed n-3 PUFA-enriched eggs. 

This research did not confirm a significant impact of the consumption of omega-3 eggs on human health, but, considering that all biochemical indicators in the blood of the subjects were within the reference values, we can say that no negative impact on human health was found. Therefore, it is necessary to undertake further research into this topic by designing dietary protocols for examinees in such a way to reduce the number of eggs consumed daily by each examinee and to extend the experimental period of egg consumption.

## Figures and Tables

**Table 1:** Chemical composition of feeding mixtures fed to laying hens (%).

Ingredient	Control K	Group P1	Group P2
Corn	48.86	48.86	48.86
Soybean cake	20.67	20.67	20.67
Roasted soybean	4.00	4.00	4.00
Sunflower cake	5.00	5.00	5.00
Alfalfa	1.50	1.50	1.50
Calcium granules	10.67	10.67	10.67
Monocalcium phosphate	1.33	1.33	1.33
Yeast	0.50	0.50	0.50
Salt	0.33	0.33	0.33
Acidifier	0.33	0.33	0.33
Nanofeed	0.33	0.33	0.33
Methionine	0.15	0.15	0.15
Premix *	1.33	1.33	1.33
Soybean oil	5.00	-	-
Fish oil	-	1.50	2.00
Linseed oil	-	3.50	3.00
Total	100.00	100.00	100.00

* One kg of premix contains: vit. A 840.000 IJ; vit. D3 210.000 IJ; vit. E 8.350 mg; vit. K3 168 mg; vit. B1 150 mg; vit B2 374 mg; vit. B6 200 mg; vit. B12 918 mg; vit. C 1.860 mg; niacin 2.100 mg; pantothenic acid 584 mg; folic acid 75 mg; biotin 7 mg; choline chloride 33.600 mg; iron 2.520 mg; iodine 76 mg; copper 425 mg; manganese 5.640 mg; zinc 175 mg; selenium 32 mg; canthaxanthin 260 mg.

**Table 2:** Fatty acid profile in laying hens’ feeding mixtures (% of total fatty acids).

Fatty Acid	K	P1	P2	*p* Value
Myristic C14:0	n.d.	1.26 ± 0.05 ^b^	1.51 ± 0.01 ^a^	0.001
Pentadecanoic C15:0	n.d.	0.17 ± 0.00 ^b^	0.20 ± 0.00 ^a^	0.001
Palmitic C16:0	11.27 ± 0.21 ^a^	10.82 ± 0.18 ^b^	11.09 ± 0.02 ^ab^	0.038
Heptadecanoic C17:0	n.d.	0.21 ± 0.1 ^b^	0.23 ± 0.01 ^a^	0.001
Stearic C18:0	4.71 ± 0.02 ^a^	4.11 ± 0.03 ^b^	4.03 ± 0.01 ^c^	0.001
Arachidonic C20:0	0.41 ± 0.00 ^a^	0.27 ± 0.00 ^b^	0.27 ± 0.00 ^b^	0.001
Behenic C22:0	0.33 ± 0.16	n.d.	n.d.	-
∑ SFA	16.72 ± 0.22 ^b^	16.84 ± 0.21 ^b^	17.33 ± 0.01 ^a^	0.006
Palmitoleic C16:1	n.d.	1.52 ± 0.03 ^b^	1.80 ± 0.01 ^a^	0.001
Octadecanoic C18:1	26.24 ± 0.02 ^a^	21.55 ± 0.07 ^b^	21.01 ± 0.13 ^c^	0.001
Eicosenoic C20:1	0.23 ± 0.05 ^c^	1.31 ± 0.03 ^b^	1.59 ± 0.01 ^a^	0.001
Erucic C22:1	n.d.	1.51 ± 0.05 ^b^	1.88 ± 0.02 ^a^	0.001
∑MUFA	26.47 ± 0.07 ^a^	25.89 ± 0.13 ^b^	26.28 ± 0.09 ^a^	0.001
Linoleic C18:2 n-6	51.84 ± 0.11 ^a^	23.23 ± 0.22 ^b^	22.54 ± 0.19 ^c^	0.001
Eicosadienoic C20:4 n-6	n.d.	0.17 ± 0.01 ^b^	0.25 ± 0.01 ^a^	0.001
∑n-6 PUFA	51.84 ± 0.11 ^a^	23.40 ± 0.21 ^b^	22.79 ± 0.19 ^c^	0.001
α-linolenic C18:3 n-3	5.04 ± 0.03 ^c^	28.47 ± 0.02 ^a^	26.33 ± 0.21 ^b^	0.001
Eicosapentaenoic C20:5 n-3	n.d.	1.86 ± 0.02 ^b^	2.42 ± 0.02 ^a^	0.001
Docosahexaenoic C22:6 n-3	n.d.	3.53 ± 0.06 ^b^	4.84 ± 0.04 ^a^	0.001
∑n-3 PUFA	5.04 ± 0.03 ^b^	33.86 ± 0.28 ^a^	33.59 ± 0.28 ^a^	0.001
n-6/n-3 PUFA	10.28 ± 0.05 ^a^	0.69 ± 0.02 ^b^	0.68 ± 0.01 ^b^	0.001

K = 5% soybean oil, P1 = 1.5% fish oil + 3.5% linseed oil, P2 = 2% fish oil + 3% linseed oil; $\bar{x}$ = mean value; sd = standard deviation; Average values in rows marked by superscript ^a,b,c^ differ significantly at *p* < 0.05, *p* < 0.01, and *p* < 0.001. SFA—saturated fatty acids, MUFA—monounsaturated fatty acids, PUFA—polyunsaturated fatty acids. n.d.—not determined.

**Table 3:** Fatty acid profile in egg yolks (mg FA/100 g of edible part of egg).

Fatty Acid	K	P1	P2	*p* Value
Myristic C14:0	16.55 ± 1.64 ^b^	25.18 ± 1.16 ^a^	25.44 ± 2.10 ^a^	0.001
Pentadecanoic C15:0	3.39 ± 0.88 ^b^	5.74 ± 0.96 ^a^	6.13 ± 1.07 ^a^	0.002
Palmitic C16:0	1455.86 ± 52.70 ^b^	1572.55 ± 46.11 ^a^	1551.85 ± 44.88 ^a^	0.005
Heptadecanoic C17:0	13.52 ± 2.76	15.57 ± 1.51	16.77 ± 1.72	0.081
Stearic C18:0	575.15 ± 41.73	562.58 ± 16.57	552.03 ± 24.16	0.482
Heneicosanoic C21:0	15.67 ± 5.05 ^a^	7.18 ± 1.10 ^b^	6.42 ± 0.49 ^b^	0.001
SFA	2080.14 ± 79.76 ^b^	2188.80 ± 40.88 ^a^	2158.64 ± 51.65 ^ab^	0.036
Myristoleic C14:1	1.46 ± 0.42 ^b^	3.89 ± 0.89 ^a^	3.32 ± 0.21 ^a^	0.001
Palmitoleic C16:1	106.86 ± 19.47 ^b^	194.86 ± 13.58 ^a^	187.07 ± 9.91 ^a^	0.001
cis-10-heptadecenoic C17:1	10.71 ± 1.29 ^b^	16.86 ± 1.53 ^a^	18.35 ± 2.09 ^a^	0.001
Oleic C18:1 cis + trans	2502.17 ± 72.02 ^b^	2700.72 ± 113.39 ^a^	2734.25 ± 102.08 ^a^	0.005
cis-11-eicosenoic C20:1	13.29 ± 2.07	16.02 ± 1.71	15.29 ± 1.89	0.102
MUFA	2634.49 ± 72.23 ^b^	2932.35 ± 103.27 ^a^	2958.31 ± 95.32 ^a^	0.001
Linoleic C18:2 n-6	1408.22 ± 216.61 ^a^	1057.95 ± 48.28 ^b^	1079.92 ± 60.96 ^b^	0.002
γ-linolenic C18:3 n-6	7.32 ± 1.08	n.d.	n.d.	-
Eicosadienoic C20:2 n-6	10.46 ± 2.03	8.17 ± 1.34	8.32 ± 1.07	0.064
Arachidonic C20:4 n-6	127.51 ± 10.25 ^a^	61.59 ± 5.91 ^b^	61.58 ± 5.88 ^b^	0.001
∑n-6 PUFA	1553.51 ± 225.91 ^a^	1127.71 ± 52.27 ^b^	1149.82 ± 66.57 ^b^	0.002
α-linolenic C18:3 n-3	65.48 ± 17.54 ^b^	370.27 ± 42.25 ^a^	358.21 ± 41.41 ^a^	0.001
EPA	n.d.	29.14 ± 3.72	31.06 ± 1.86	0.462
DHA	84.40 ± 8.96 ^b^	199.18 ± 18.17 ^a^	191.44 ± 24.74 ^a^	0.001
∑n-3 PUFA	149.88 ± 26.34 ^b^	598.59 ± 46.60 ^a^	580.71 ± 57.44 ^a^	0.001
∑n6/∑n3 PUFA	10.36 ^a^	1.88 ^b^	1.98 ^b^	0.001

K = 5% soybean oil, P1 = 1.5% fish oil + 3.5% linseed oil, P2 = 2% fish oil + 3% linseed oil; $\bar{x}$ = mean value; sd = standard deviation; Average values in rows marked by superscript ^a,b^ differ significantly at *p* < 0.05 and *p* < 0.01. SFA—saturated fatty acids, MUFA—monounsaturated fatty acids, PUFA—polyunsaturated fatty acids. n.d.—not determined.

**Table 4:** Egg health indexes.

Indicator	K	P1	P2
Atherogenic index (AI)	0.498	0.471	0.470
Thrombogenic index (TI)	0.833	0.555	0.556
Hypo/hypercholesterolemic index (HHI)	2.755	2.843	2.824

K = 5% soybean oil; P1 = 1.5% fish oil + 3.5% linseed oil, P2 = 2% fish oil + 3% linseed oil.

**Table 5:** Effects of consumption of conventional eggs (K group) and n-3 PUFA-enriched eggs (P1 group) on the profile of fatty acids in examinees’ blood serum.

Fatty Acid	K	P1	*p* Value
Myristic, C14:0	29.72 ± 8.75	38.63 ± 10.25	0.136
Palmitic, C16:0	796.23 ± 145.7 ^a^	533.93 ± 210.8 ^b^	0.031
Stearic, C:18:0	219.34 ± 33.89	157.10 ± 69.39	0.076
∑SFA	1045.31 ± 181.4 ^a^	729.68 ± 275.9 ^b^	0.041
Palmitoleic, C16:1	57.81 ± 23.4	46.67 ± 18.1	0.379
Octadecanoic, C18:1	534.54 ± 121.2	377.98 ± 190.5	0.120
∑MUFA	592.26 ± 139.8	424.65 ± 206.1	0.130
Linoleic, C18:2 n-6	1013.21 ± 101.3	869.22 ± 322.3	0.321
γ-linolenic, C18:3 n-6	18.91 ± 4.0	16.96 ± 4.8	0.487
Eicosadienoic, C20:2	7.21 ± 0.5	7.93 ± 0.4	0.324
Dichomo-gamma-linolenic, C20:3n-6	50.44 ± 12.3 ^a^	30.94 ± 9.67 ^b^	0.012
Arachidonic, C20:4 n-6	359.49 ± 79.1 ^a^	234.32 ± 94.2 ^b^	0.031
∑n-6 PUFA	1449.26 ± 155.1	1159.37 ± 427.7	0.142
α-linolenic, C18:3 n-3	10.39 ± 2.3 ^b^	16.18 ± 3.1 ^a^	0.010
Eicosapentaenoic, C20:5 n-3	10.11 ± 2.0	12.49 ± 3.4	0.264
Docosahexaenoic, C22:6 n-3	44.94 ± 10.7	43.28 ± 19.45	0.858
∑n-3 PUFA	65.44 ± 14.3	71.95 ± 29.4	0.861

K = 5% soybean oil; P1 = 1.5% fish oil + 3.5% linseed oil. Average values in rows marked by superscript ^a,b^ differ significantly at *p* < 0.05.

**Table 6:** Arterial blood pressure of examinees ($\bar{x}$ ± sd).

Group/ Indicators	K		*p* Value	P1		*p* Value
	Before	After		Before	After	
n	10			10		
SBP, mmHg	104.2 ± 16.7	104.8 ± 13.4	0.927	108.1 ± 11.2	106.6 ± 12.1	0.785
DBP, mmHg	68.2 ± 11.4	67.6 ± 4.3	0.873	73.4 ± 6.6	73.7 ± 6.7	0.917
MAP, mmHg	80.2 ± 11.7	80.0 ± 6.5	0.961	85.0 ± 5.8	85.1 ± 6.2	0.965

$\bar{x}$ = mean value; sd = standard deviation; K = 5% soybean oil; P1 = 1.5% fish oil + 3.5% linseed oil; SBP—systolic blood pressure; DBP—diastolic blood pressure; MAP—mean arterial pressure; Before = start of the experiment involving examinees who consumed eggs and assessment of such consumption of health indicators; After = end of the experiment involving examinees who consumed three boiled eggs of the L class over three weeks.

**Table 7:** Effects of consumption of conventional (K) and n-3 PUFA-enriched eggs (P1) on examinees’ blood biochemical indicators ($\bar{x}$ ± sd).

Indicator	K		P1	
	Before	After	Before	After
Leukocytes (10^9^/L)	7.02 ± 1.32	7.40 ± 2.71	7.34 ± 2.32	7.23 ± 0.99
Erythrocytes (10^12^/L)	4.77 ± 0.34	4.85 ± 0.39	4.85 ± 0.48	4.81 ± 0.50
Hemoglobin g/L	142.56 ± 14.21	143.78 ± 15.42	144.90 ± 14.12	143.70 ± 14.82
Hematocrit (L/L)	0.40 ± 0.03	0.41 ± 0.04	0.42 ± 0.04	0.40 ± 0.04
MCV (fl)	83.32 ± 2.61	83.57 ± 3.60	85.46 ± 1.80	83.87 ± 2.51 *
MCH (pg)	29.89 ± 1.67	29.59 ± 1.45	29.96 ± 0.69	29.92 ± 0.78
MCHC (g/L)	358.56 ± 14.16	354.44 ± 6.97	349.60 ± 5.70	356.70 ± 2.79 *
RDW-CV (%)	12.93 ± 0.91	12.78 ± 0.68	12.67 ± 0.45	12.72 ± 0.42
Platelets (10^9^/L)	269.11 ± 62.30	268.70 ± 57.90	251.62 ± 62.69	245.70 ± 54.29
MPV (fl)	8.32 ± 1.46	8.68 ± 1.73	10.45 ± 1.03	8.30 ± 1.11 *
Erythrocytes sedimentation (mm/3.6 KS)	8.67 ± 8.63	9.67 ± 10.14	7.20 ± 4.44	5.80 ± 4.10
Fibrinogen (g/L)	3.01 ± 0.75	3.01 ± 0.62	2.98 ± 0.74	2.65 ± 0.59
Glucose (mmol/L)	4.99 ± 0.37	4.86 ± 0.28	4.96 ± 0.69	4.93 ± 0.69
Urea (mmol/L)	4.74 ± 1.78	5.10 ± 1.60	5.68 ± 1.33	5.25 ± 1.23
Creatinine (μmol/L)	79.22 ± 17.29	77.11 ± 16.10	80.10 ± 19.34	76.80 ± 17.31
Urates (μmol/L)	305.56 ± 85.16	278.67 ± 59.17	329.6 ± 75.98	302.20 ± 76.10 *
hsCRP (mg/L)	3.28 ± 3.33	3.40 ± 4.29	2.15 ± 2.24	1.34 ± 1.02
Iron (μmol/L)	15.02 ± 5.42	13.00 ± 6.78	17.14 ± 8.45	20.55 ± 7.07
Transferrin (g/l)	2.59 ± 0.43	2.68 ± 0.10	2.59 ± 0.44	2.66 ± 0.48
Cholesterol (mmol/L)	4.80 ± 0.87	4.63 ± 0.68	5.10 ± 0.69	5.24 ± 0.86
Triglycerides (mmol/L)	0.96 ± 0.35	0.97 ± 0.39	0.91 ± 0.30	0.88 ± 0.25
HDL-cholesterol (mmol/L)	1.53 ± 0.38	1.48 ± 0.43	1.43 ± 0.31	1.45 ± 0.36
LDL-cholesterol (μmol/L)	2.80 ± 0.60	2.69 ± 0.59	3.14 ± 0.54	3.13 ± 0.62
HDL-C/cholesterol (%)	32.56 ± 9.49	32.11 ± 9.66	28.30 ± 5.77	28.10 ± 7.36

$\bar{x}$ = mean value; sd = standard deviation; K = 5% soybean oil; P1 = 1.5% fish oil + 3.5% linseed oil; Before = start of the experiment involving examinees who consumed eggs and assessment of such consumption of health indicators; After = end of the experiment involving examinees who consumed three boiled eggs of the L class over three weeks. Average values in rows within group marked by * differ significantly at *p* < 0.05.

## Data Availability

The data used to support the findings of this study can be made available by the corresponding author upon request.
